# Global barriers to decision makers for prioritizing interventions for obesity

**DOI:** 10.1038/s41366-024-01650-z

**Published:** 2024-10-16

**Authors:** Lars Holger Ehlers, Nicoline Weinreich Reinstrup, Renée Hangaard Olesen, Jens-Christian Holm, Phil McEwan, Carel W. Le Roux

**Affiliations:** 1 Nordic Institute of Health Economics, Aarhus, Denmark; 2 University Hospital Holbæk, Holbæk, Denmark; 3 Health Economics and Outcomes Research Ltd, Cardiff, UK; 4 University College Dublin, School of Medicine, Dublin, Ireland

**Keywords:** Health policy, Public health

## Abstract

The treatment of obesity remains underprioritized. New pharmacologic options for the treatment of obesity have shown effectiveness and safety but are not widely reimbursed. Despite the unmet need and the existence of effective prevention and treatment strategies, substantial barriers exist to effectively address obesity as a disease. The purpose of this scoping review was to investigate the barriers for decision makers in prioritizing interventions for obesity and to seek out interconnection between barriers to prevention and treatment. A scoping review was conducted using a systematic search of both scientific databases and Health Technology Assessment (HTA) databases. Studies that addressed barriers to reimbursement or prioritization of obesity treatment and prevention were included. A total of 26 articles and 14 HTAs were included. Four main barriers for decision makers to prioritize new interventions for obesity were identified: perceptions, knowledge, economics, and politics. There was a high degree of interconnectedness among barriers, as well as large overlaps between barriers in relation to bariatric surgery, pharmacologic treatments, and prevention regulation. Multiple barriers exist that impact decision makers in prioritizing interventions for treating obesity. A strong interconnectedness of the barriers was found, indicating a systems approach to improve global prioritization to address the disease. This study suggests that decision makers should carefully consider all main barriers when addressing the obesity epidemic.

## Introduction

Obesity has emerged as a significant global health challenge, reaching epidemic proportions in many parts of the world [1–3]. Every country is affected by obesity, with some lower-income countries showing the highest increases. No country has managed to show a decline in prevalence across their entire population [1, 3].

The consequences for people living with obesity are increased risk of chronic obesity-related complications, such as type 2 diabetes, cardiovascular disease, and several forms of cancer, resulting in poorer quality of life, lower education and income, and premature death [2–5]. The global economic impact of obesity is expected to surpass USD 4 trillion by 2035, with 1 in 4 people (nearly 2 billion) living with obesity by 2035 if current trends prevail, indicating a great need for measures to prevent obesity to save societal resources [3, 6].

Action on obesity is commonly siloed and fragmented, and obesity remains underprioritized within global health and national healthcare strategies as a risk factor rather than a disease in its own right [2, 3, 7]. Preventive efforts have seen limited success and are insufficient in reversing the increasing obesity incidence [3, 7, 8]. Bariatric surgery rapidly and substantially reduces weight and risk of obesity-related complications but remains difficult to scale, with most countries reporting limitations in current capacity levels [9, 10]. New pharmacologic options have been shown to be effective and safe but are currently not reimbursed in most countries, limiting availability to select subgroups of patients and/or patients able to pay out of pocket [11, 12].

In 2035, more than half the global population will have a body mass index (BMI) above 25 kg/m^2^ if prevention, treatment, and support do not improve [3]. Even though effective prevention and treatment strategies exist, barriers may prevent actions to address the disease of obesity [2, 3, 13]. The projected increase in obesity and the current barriers to treatment raise several questions: (1) Can we afford inaction in the worldwide implementation of obesity prevention policies?; (2) Why are there not more healthcare resources allocated to address the disease of obesity?; and (3) Why are effective pharmaceutical treatments not reimbursed in all countries?

International recommendations for addressing obesity suggest coordinated efforts rather than prioritizing single technologies [2, 3, 13, 14]. This involves multiple decision makers at different levels of government as well as decision makers in and outside of healthcare institutions [2, 3, 13, 14]. To this end, we conducted a scoping review to investigate the barriers to decision makers in prioritizing interventions for obesity, while seeking out interconnection between barriers to prevention and treatment. An overall analytical perspective was applied including bariatric surgery, pharmacologic treatment, and regulation (state intervention e.g., sugar tax or marketing restrictions), analyzing barriers at the local, national, and international levels, with results summarized across various scientific disciplines.

## Methods

### Study design

A scoping review was chosen due to the complexity of the topic and in order to provide a comprehensive overview of currently available evidence. The scoping review followed the methodologic framework described by Peters et al. [15] and enhanced by Mak et al. [16].

#### Information sources and search strategy

A comprehensive search strategy was developed in accordance with standard practice guidelines as described by Aromataris et al. [17]. The literature search was two-fold, including a systematic literature search in academic databases and a search in Health Technology Assessment (HTA) databases.

The academic databases search was conducted in PubMed, Scopus, and ProQuest. All three databases were searched for eligible literature on January 15, 2024. The search was conducted as a block search with three search term groups pertaining to the themes of overweight and obesity, healthcare decision makers, and reimbursement. Keywords and subject headings were informed by existing literature relevant to the areas of interest, identified through an initial literature search, and supplemented with relevant synonyms. The search was limited to include literature published between 2014 and 2024, and was restricted to full text literature in English, Danish, Swedish, and Norwegian. The detailed search strategy is presented in Appendix 1.

The HTA database search included nice.org.uk, cadht.ca, tlv.se, janusinfo.se, fhi.no, nyemetoder.no, sst.dk, masc.gov.au, pbs.gov.au, and pharmac.govt.nz. All searches in HTA databases were conducted with the theme overweight and obesity, using search terms relevant for the specific database, such as “fetma” in Swedish databases.

#### Study selection process

All literature identified from the searches was aggregated using the software Rayyan, and duplicates were removed. Two independent researchers first screened titles and abstracts. Potentially relevant sources were then retrieved in full text and screened for the inclusion and exclusion criteria by one or more reviewers. Disagreements throughout the screening process were discussed and resolved between the two reviewers, with the involvement of a third reviewer during full-text screening.

#### Inclusion and exclusion criteria

Studies were eligible for inclusion if they addressed barriers to reimbursement or prioritization of bariatric surgery, pharmacologic treatments, or regulation aimed at obesity prevention. There were no restrictions on the target population of the obesity intervention, thus encompassing all BMI groups, ages, genders, and ethnicities. To qualify for inclusion, studies had to examine barriers from the perspective of decision makers with influence on reimbursement and prioritization of obesity interventions, such as policymakers and HTA bodies. Only studies that specifically discussed barriers to reimbursement and prioritization were considered. While we acknowledge that barriers to implementation and prioritization may be interconnected, studies focusing solely on barriers to implementation or barriers to effective treatment at the clinical level were excluded, as different stages in the policy process will be subject to different barriers [18]. No specific exclusion criteria regarding study design were set. Location or country of the obesity intervention, as well as the type of intervention, were not restricted. Therefore, interventions for both prevention and treatment of obesity were considered, spanning various approaches including medication, surgery, dietary interventions, sugar taxes, and public policies.

### Analysis

#### Data extraction

Full-text articles and decision reports from national HTA organizations (HTA decision reports) that met the inclusion criteria were charted to summarize the findings. The elements chosen for data extraction were based on Micah et al. [15] and tailored to suit the objectives of this scoping review. The elements included the main study characteristics (authors, title, year of publication, aim/purpose), as well as geographical location of the obesity intervention, type of intervention, setting of intervention, target population, reported barriers to reimbursement of obesity interventions, and future recommendations. The data extraction has been summarized in Appendix 2.

#### Data summary and synthesis

Barriers to reimbursement of obesity interventions have been identified through thematic analysis in accordance with Braun and Clarke [19]. Three reviewers conducted the thematic analysis. Initial codes were generated independently and modified iteratively throughout the analysis. Once initial coding had been conducted, codes were grouped together narratively to identify and contextualize main barriers and subthemes.

A quantitative descriptive analysis was conducted to examine the frequency of the barriers and their interconnections in all primary literature, and results were summarized as absolute and relative frequencies. This was done separately for bariatric surgery, pharmacologic treatment, and regulation, as differences in the types of barriers or subthemes were expected. Only literature from the systematic literature search was included in the quantitative analysis, as the inclusion of HTA decision reports was deemed likely to bias the results.

## Results

### Literature search

**Fig. 1:**
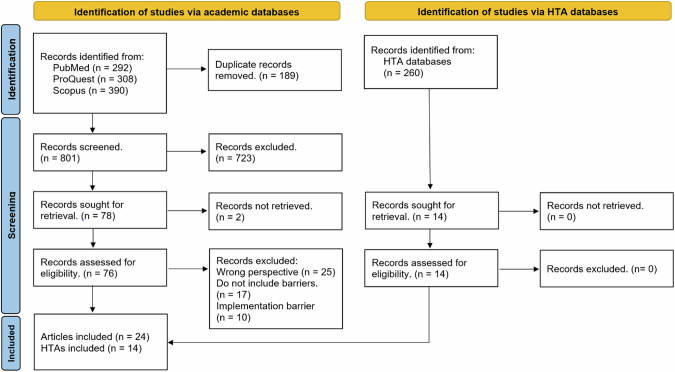
PRISMA diagram illustrating study and HTA screening process.

A total of 990 articles were identified through the database search in PubMed, Scopus, and ProQuest. Following removal of duplicates and screening of titles and abstracts, 78 full-text articles were retrieved, and 24 were ultimately included. The search of HTA databases identified 260 reports, of which 14 were included (Fig. 1). In total, 38 articles and HTA decision reports were included for data extraction.

The primary literature was conducted in multiple countries, a multitude of different settings, and multiple types of obesity interventions. Included articles were published between 2014 and 2023. Most studies were conducted in western high-income countries (*n* = 20, 83%), with the remaining 4 (17%) studies being conducted in Brazil, China, Mexico, and Fiji. Of the 24 included studies, the most frequently occurring country was the USA (*n* = 12, 50%), followed by Canada and England, with 3 (13%) articles conducted in each. The studies addressed pharmacologic treatment (*n* = 11, 46%), bariatric surgery (*n* = 10, 42%), and regulation (*n* = 19, 79%), with 10 (42%) studies addressing two or more types of obesity interventions.

HTA decision reports were mainly from England (*n* = 7, 50%), with the remaining reports from Canada, Australia, Denmark, and Sweden, due to the selection of HTA databases. Four (29%) of the HTA decision reports addressed bariatric surgery, while the remaining 10 (71%) addressed pharmacologic treatment. The search did not identify reports prior to 2013, although there was no restriction on publication date.

### Main barriers

This scoping review identified four main barriers for decision makers in prioritizing new interventions for obesity. *Perceptions* reflect barriers stemming from the lack of common understanding and acknowledgement of obesity as a chronic disease among stakeholders and in society. *Knowledge* addresses barriers due to gaps in the evidence base and limitations in the spread of knowledge of effective obesity intervention strategies. *Economics* concerns affordability issues due to the large and increasing prevalence of obesity worldwide, uncertainty about the long-term consequences of obesity interventions, and wider economic consequences beyond health sector economics. *Politics* encompasses the lack of popularity, conflicting stakeholder interests, and the difficulties of policymaking in a complex area.

#### Perceptions

##### Formal recognition of obesity as a disease

Countries such as the USA and Canada and various organizations including the World Health Organization and the European Parliament have formally recognized obesity as a chronic disease, while other countries and organizations have not. The lack of formal recognition by governments, national health authorities, and healthcare institutions is a barrier for national healthcare providers to prioritize services and funding, and can affect the type of interventions and approaches implemented by governments or covered by private and public health plans [20–23]. An example is China, where the lack of formal recognition by health authorities means that medications for the disease of obesity are not covered by medical insurance, and is also described as a reason for the regional variations in bariatric and metabolic surgery [24]. Another example is the UK, where this same lack of formal recognition by the National Health Service and local health authorities causes severe capacity constraints in bariatric surgery and a relatively low level of state-funded bariatric surgery procedures per year [21].

##### Decision maker acknowledgement

Even with formal recognition from national authorities, decision makers and stakeholders across the health sector tend to have different understandings and beliefs. Lack of decision maker acknowledgement represents a barrier to prioritizing interventions for treating the disease of obesity despite formal declarations and health laws, as such lack of acknowledgement can slow down or stop decision making and local implementation [25]. In the USA, the Patient Protection and Affordable Care Act of 2010 and the American Medical Association’s formal recognition of obesity as a disease in 2013 were significant developments in US healthcare policy; however, the geographic variation in the interpretation and implementation across the USA may reflect variability in acknowledgement, as well as other constraints limiting local health policy adoption and development [26–29]. Similarly, the lack of decision maker acknowledgement of obesity as a disease may limit local interventions for childhood obesity and school wellness policies in the USA [30]. In Canada, the Canadian Medical Association declared obesity a chronic disease in 2015, but 2 years after the declaration none of the provincial or territorial governments had officially recognized obesity as a chronic disease [20].

##### Common understanding

Population surveys from different parts of the world indicate that most people still view obesity as a risk factor for other diseases rather than a disease itself, and most people believe obesity is self-inflicted [25, 26, 31, 32]. In a Canadian study, the lack of common understanding of obesity as a chronic disease by Canadian public and private payers, health systems, employers, and the public was described as having a “trickle-down effect” on access to evidence-based intervention resources [20]. The dominant cultural narrative around obesity in society can even fuel assumptions about personal irresponsibility that project blame and shame upon individuals living with obesity. Obesity stigma and weight bias can lead to discrimination and affect decision maker support for interventions [20, 33]. Even among healthcare professionals who treat people living with obesity, there are different understandings of obesity and multiple barriers to the implementation of effective interventions [26]. Furthermore, there are disagreements among social scientists about the characterization of obesity as a disease, whether obesity justifies government intervention, and to what degree obesity is a matter for the healthcare sector [34]. This is part of a bigger discussion about the regulation of markets (foods, alcohol, drugs, etc), as well as behaviors such as gambling, and it addresses the question of the appropriate point of equilibrium between free choice and state intervention (regulation), as well as the question of when risks can be considered to be acceptable or tolerable [34].

#### Knowledge

##### Gaps in the evidence base

Evidence is vital for decision makers to understand where and how to intervene. Gaps in the evidence base about obesity and the effects of interventions lead to barriers to decision-makers’ prioritization of interventions for obesity. Such gaps also tend to weaken arguments for intervention, as stakeholders with different interests may introduce opposing evidence and frame agendas differently [20, 21, 24, 35]. A lack of evidence of the long-term effects of pharmaceutical interventions, especially evidence of the effect on obesity-related complications, is mentioned in HTA organizations’ rejections of national reimbursement submissions [36–47]. The need for evidence that clearly identifies the causes of obesity and specific areas for intervention is described as a vocal point for prioritizing prevention regulation initiatives. Some articles call for a better understanding of the wider determinants of health, including deprivation and the broader environment to remove responsibility from the individual and apply a holistic approach to prevention regulation [25, 31, 48]. There is a greater lack of evidence of the effectiveness of pharmaceutical and surgical interventions in certain populations. For example, populations of Asian descent have a higher body fat percentage, more cardiovascular risk factors, and a higher all-cause mortality rate compared with Caucasians. Consequently, a study from China highlights the need for high-quality evidence on the effectiveness of pharmacologic treatment and surgical interventions in Chinese populations [24].

##### Knowledge dissemination

Even in areas supported with solid evidence, there may be barriers to knowledge dissemination for decision makers [28, 33, 48–51]. Studies in the USA, Canada, Mexico, and Fiji show the importance of knowledge brokers, lobbyists, and policy entrepreneurs in knowledge dissemination to politicians [28, 33, 48, 50, 51]. Research is mainly written by researchers for researchers and is thus generally difficult for non-experts to understand [25]. Gaps in medical curricula and postgraduate education programs may lead to lack of updated knowledge, lack of training to address obesity, and failure to communicate evidence-based knowledge to patients and caregivers [21, 32]. Historical safety issues and market withdrawals of pharmacologic treatments for obesity may constitute barriers to knowledge dissemination and influence physicians’, patients’, lay peoples, and decision makers’ expectations of the effectiveness and safety of modern pharmacologic treatment [29].

#### Economics

##### Affordability

The high prevalence of obesity questions the affordability due to the large numbers of people living with obesity requiring healthcare services. This places strain on both public healthcare budgets and private insurance schemes, raising concerns about sustainability and equity in access [21, 23, 27–29]. The problem of affordability is especially raised in the papers and HTA decision reports on pharmacologic treatment and bariatric surgery [29, 32, 37, 38, 43, 47]. In the USA, the Affordable Care Act of 2010 included provisions for private and public health insurance plans that expanded coverage for lifestyle/behavior modification and bariatric surgery for the treatment of obesity. However, this was introduced with restrictions on consumer cost-sharing and no premium surcharges for having obesity. Furthermore, pharmacologic treatment was not included [23, 28, 29]. Affordability can also be a barrier to prevention interventions when choices must be made regarding the allocation of scarce budget resources. A survey among US state representatives and senators showed that budgetary constraints were at the forefront of decision makers’ opinions, making funding of obesity prevention policies difficult [30].

##### Uncertainty

Uncertainty about the extent of the economic burden of obesity (i.e., the direct and indirect costs of obesity) now and in the future is a barrier, as it makes the need for interventions unclear [21, 52]. Uncertainty about the cost-effectiveness and budget impact of interventions makes it more difficult for decision makers to prioritize [20, 22, 32, 52]. Several HTA decision reports from the UK, Canada, Australia, and the Scandinavian countries describe uncertainty about the cost-effectiveness of pharmacologic treatments as a reason for not reimbursing new effective treatments for obesity [36–40, 43, 44, 46, 53]. The cost-effectiveness of regulatory prevention of obesity is generally under-investigated and very uncertain, with current health economic models suboptimal to allocate a utility gain to weight loss of 20 to 30% [20].

##### Wider economic aspects

Decision makers often have to take into account the bigger economic picture, whether it is the national economy or the government’s or healthcare system’s budget [50, 52, 54–56]. In Australia, the economic burden of obesity was presented as an argument for generating policies for regulatory interventions targeting obesity prevention [52, 55]. This “economic rationale” received wide attention in 2006 and 2008 when figures from modeling reports commissioned by Diabetes Australia were used by parliamentarians. However, the food, beverage, and advertising industries successfully managed to create a political focus on the wider economic effects of regulation that would reduce revenue and jobs, and therefore the proposed policies were not prioritized [52, 55].

#### Politics

##### Lack of popularity

Political barriers to reimbursement of obesity treatments and support of interventions include low support among voters, often due to competing priorities or the stigma associated with obesity [34, 49]. Surveys with laypeople in the USA, UK, and Germany show that respondents attribute the responsibility for obesity primarily to the individual; the same pattern was seen for alcohol and tobacco dependence, but not for depression [49]. The higher the attribution of personal responsibility, the more strongly respondents endorsed individual liability for treatment costs. Respondents judged information as the most effective and fiscal policies as the least effective [49]. Studies on political processes from Australia, Mexico, and the USA also show low priority among politicians of political regulation and a lack of civil society cohesion on regulatory prevention interventions [50, 52, 56]. Studies from the USA and Brazil on the barriers and facilitators of the adoption of obesity prevention policies found most news media coverage to be negative [31, 56].

##### Strong interests and lobbyism

Strong influences from interest groups, such as the food industry, agriculture, and trade, as well as their advocacy groups, further complicate policymaking and regulation by promoting conflicting agendas [31, 55]. An Australian study explaining resistance to regulatory interventions to prevent obesity and improve nutrition concludes that Australia is a substantial way from having the conditions in place where a tax might be successfully implemented and that regulation initiatives are facing strong, united opposition from the beverage industry, sugar industry and sugarcane growers [55]. The Brazilian study on barriers to the adoption of policies to reduce ultra-processed food consumption concluded that the main barrier was a combination of the corporate political activities of the food industry and a weak government vulnerable to commercial interests [31]. The lack of a robust scientific evidence base showing effective prevention of obesity makes it very easy for lobbying by companies with vested interests [31].

##### Difficulties of policy making

Policy formulation on obesity is complex, as it addresses the difficult task of changing behavior in people and organizations, and is influenced by diverse stakeholder interests [34, 51, 52, 55, 56]. Policy makers face challenges in formulating effective policies due to the topic of obesity falling between levels of government and institutions [54]. Furthermore, a study identified policymakers’ limited understanding of the connections between a range of policy issues and obesity [48]. A research study on using obesity research to shape obesity policy in Minnesota, USA suggests challenges to policymaking include the amount and complexity of scientific research and the limited relationships between researchers, politicians, and decision makers [35]. The framing of the obesity problem defines the political agendas and limits the interventions discussed by decision makers [22, 25, 34, 35, 48]. A case study of Fijian policy makers on the obesity prevention regulation and policy landscape describes the difficulties for policy making due to the poor economic situation, low food self-sufficiency, power inequalities, inappropriate framing of obesity, limited policy evidence, and limited resource sharing; all hampering obesity policy developments [51]. In Australia, responsibility for obesity prevention regulation sits across all levels of government and several intergovernmental institutions, creating a complicated policy space and making health policy challenging in the absence of national leadership and funding [54]. Furthermore, in Australia, the political arguments against regulatory interventions targeting obesity prevention are often characterized by a libertarian/neo-libertarian rhetoric emphasizing individual responsibility, and a negative view of freedom (as free from “nanny-state” intervention) [52].

### Overlaps and interconnectedness of barriers

The quantitative analysis of the barriers revealed large overlaps between barriers discussed in relation to bariatric surgery, pharmacologic treatments, and regulation (Table 1). Barriers for bariatric surgery and for pharmacologic treatment are almost identical. Furthermore, barriers related to perceptions and knowledge are almost identical for bariatric surgery, pharmacologic treatment, and regulation to promote prevention of obesity. HTA decision reports were not included in the quantitative analysis; however, a barrier in all HTA decision reports was knowledge, while economics was also a barrier in most HTA decision reports.

**Table 1:** Frequency of barriers described in relation to bariatric surgery, pharmacologic treatments, and obesity regulation.

Barrier	Subtheme	Pharmacologic treatment (*n* = 11)^a^	Bariatric surgery (*n* = 10)^a^	Regulation (*n* = 19)^a^
Perceptions	Formal recognition of obesity as a disease	8 (73%)	8 (80%)	5 (26%)
	Decision maker acknowledgement	9 (82%)	6 (60%)	10 (52%)
	Common understanding	6 (54%)	6 (60%)	13 (68%)
Knowledge	Gaps in the evidence base	3 (27%)	3 (30%)	6 (31%)
	Knowledge dissemination	7 (64%)	8 (80%)	12 (63%)
Economics	Affordability	4 (36%)	7 (70%)	2 (11%)
	Uncertainty	3 (27%)	3 (30%)	3 (16%)
	Wider economic aspects	0 (0%)	0 (0%)	7 (37%)
Politics	Lack of popularity	2 (18%)	2 (20%)	7 (37%)
	Strong interest and lobbyism	0 (0%)	0 (0%)	8 (42%)
	Difficulties of policy making	2 (18%)	2 (20%)	12 (63%)

^a^*n* refers to the number of articles.

**Fig. 2:**
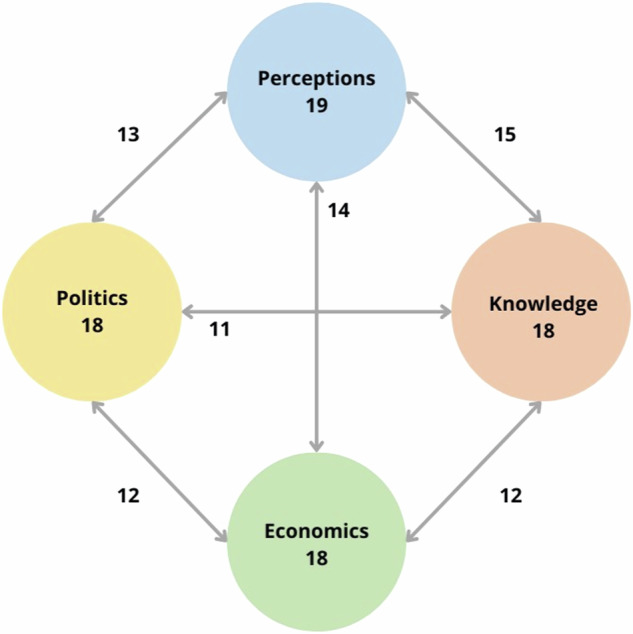
Frequency and interconnectedness of barriers*. *Numbers in circles illustrate the number of articles mentioning each type of barrier. Numbers associated with arrows illustrate the number of times different barriers are described together.

The quantitative analysis showed a high degree of interconnectedness among barriers (Fig. 2). None of the papers discuss single barriers, but always two or more types of barriers together (mean number of barriers addressed per paper was 3.0). All of the main types of barriers seem to be important for all types of interventions. Differences in subthemes were seen among bariatric surgery, pharmacologic treatments, and regulation. “Strong interest groups” and “wider economic aspects” were only discussed in terms of regulations.

## Discussion

This scoping review summarizes the evidence of global barriers to decision makers’ prioritization of interventions for the disease of obesity. We identified barriers in terms of *Perceptions* reflecting the lack of common understanding and acknowledgement of obesity as a chronic disease, *Knowledge* in addressing the gaps in the evidence base and limitations in the spread of knowledge of effective obesity intervention strategies, *Economics* concerning affordability issues, uncertainty, and wider economic consequences, and *Politics* encompassing the lack of popularity, conflicting stakeholder interests, and the difficulties of policy making in a complex area. To the best of our knowledge this is the first review providing an overall view of the barriers addressing bariatric surgery, pharmacologic treatment, and regulation to improve the prevention and treatment of obesity that includes both health and social sciences.

The main strength of our study is the overall aggregation and separation of main barriers and subthemes for bariatric surgery, pharmacologic treatment, and regulation to improve the treatment and prevention of obesity, respectively. Furthermore, the evidence summarized is extracted across various scientific disciplines including health and social sciences globally and includes different types of literature including HTAs. The inclusion of HTAs may introduce bias, as some applicants might withdraw their applications if not approved. However, most of the included HTA organizations do not permit applicants to withdraw their submissions, making such bias limited. None of the identified barriers and subthemes were surprising on their own; however, the overlaps and the strong interconnectedness among the barriers in the diverse fields of policy regulation and healthcare is an important finding with policy implications. Notably, no study identifies only a single barrier, yet only a few studies consider all four barriers. As demonstrated by this analysis, decision-makers who focus on just one barrier are unlikely to advance the prioritization of obesity prevention and treatment successfully. This fragmented approach risks overlooking related barriers in the broader context, leading to ineffective efforts to overcome obstacles to reimbursement and prioritization.

The main limitation of the study is the limited number of studies identified. Despite obesity being one of the world’s leading health crises, only 24 scientific papers across disciplines directly addressed barriers to decision makers prioritizing obesity. However, our study differs from other reviews of barriers. Several reviews have been conducted to address barriers in the clinical setting [57, 58]. Another type of review focused on implementation barriers for health technologies [59, 60]. Both types of reviews and studies were excluded from our analysis. A notable difference between these reviews and our review lies in the recommendations following the findings. The two other types of reviews typically recommend specific initiatives directed at a single type of barrier (e.g., better education of doctors to improve their dialogue with their patients about obesity or the use of knowledge brokers to help “translate” evidence to decision makers). Our review points to the limitations of addressing barriers in isolation. Instead, our findings suggest a systems approach to addressing the global barriers to decision makers focusing on the interconnectedness of the barriers [61, 62]. For example, getting a formal recognition of obesity as a disease may prove to have a limited effect if the declaration is formulated without consideration of its possible derived effects on the related politics, economics, evidence, and perceptions. Thought should also be paid to how the disease of obesity is defined to influence the barrier concerning perception, as defining obesity as having a biological basis and in most cases as a neurological disease may prove to lessen the resistance from industry, once again illustrating the interconnectedness of the barriers [63]. Educational and communication efforts emphasizing an evidence-based understanding of obesity to improve the dialogue between the doctor and the patient may also have a limited effect without consideration of the interconnectedness of barriers and the need for alignment with the overall perceptions, knowledge, politics, and economic barriers.

The findings in this review also suggest barriers are strong given the high degree of overlaps and interconnectedness. Together, the four main barriers form a stronghold where barriers defend each other. A successful example of facing a complex problem with multiple barriers is the battle against the tobacco epidemic [64]. Smoking has been addressed through political, economic, and knowledge dissemination initiatives aimed at tackling the issue. This multifaceted approach has required addressing several barriers simultaneously, highlighting the need to tackle interconnected barriers concurrently to achieve effectiveness. Other examples include reducing high cholesterol (statins) and migraine treatment. These examples might help inspire the breaking down of barriers to obesity treatment in the future [64–66]. Furthermore, the study sought to identify overlapping and interconnected themes between barriers to prevention and treatment. Clarifying these overlaps in barriers should help inform aligned but distinctive reviews in the future.

## Conclusion

This study revealed multiple barriers to decision makers in prioritizing interventions for the disease of obesity, with the four main barriers being perceptions, knowledge, economics, and politics. The strong interconnectedness of the barriers suggests a systems approach to improve global prioritization of the fight against obesity is needed. This study suggests that decision makers should take careful considerations of all main barriers when addressing the obesity epidemic.

## Supplementary information

Appendix 1 - Search strategy

Appendix 2 - Data extraction
