# Supplementary material for: Global barriers to decision makers for prioritizing interventions for obesity
Source: Int J Obes (Lond). 2024 Oct 16;49(2):246–53. doi: 10.1038/s41366-024-01650-z (PMC11805708; doi:10.1038/s41366-024-01650-z)
Supplement: Supplementary file 1 — Appendix 1 - Search strategy [file 41366_2024_1650_MOESM1_ESM.docx]

Appendix 1: Search strategy in academic databases

| **Database** | **Date** | **Articles identified** | **Search string** |
| --- | --- | --- | --- |
| Pubmed | 15-01-2024 | 292 | (((((((((Adipositas [Title/Abstract]) OR Obesity[Title/Abstract]) OR (Overweight[Title/Abstract])) OR (Obese[Title/Abstract])) OR (overweight[MeSH Terms])) OR (BMI[Title/Abstract])) OR (body mass index[MeSH Terms])) OR (body mass index[Title/Abstract]))) AND (((((((Policy[MeSH Terms]) OR (Policy[Title/Abstract])) OR (Policymakers[Title/Abstract])) OR (government[Title/Abstract]) OR (authority[Title/Abstract]) OR (Decision-maker[Title/Abstract])) OR (health policy[MeSH Terms])) OR (health policy[Title/Abstract]) OR (HTA [Title/Abstract]) OR (health technology assessment [Title/Abstract])) AND ((((((((((((((Reimbursement[Title/Abstract]) OR (funding[MeSH Terms])) OR (Funding[Title/Abstract])) OR (budget[MeSH Terms])) OR (budget[Title/Abstract])) OR (medical expenses[Title/Abstract])) OR (healthcare costs[MeSH Terms])) OR (Healthcare costs[Title/Abstract])) OR (financial management[MeSH Terms])) OR (financial management[Title/Abstract])) OR (economics[MeSH Terms])) OR (economics[Title/Abstract])) OR (barrier[Title/Abstract])) Filters: Books and Documents, Congress, Government Publication, Guideline, Historical Article, Interview, Introductory Journal Article, Legislation, Letter, Meta-Analysis, Practice Guideline, Review, Systematic Review, Danish, English, Norwegian, Swedish, from 2014 - 2024 Sort by: Publication Date |
| Scopus | 15-01-2024 | 390 | (TITLE ("Adipositas") OR TITLE ("obese") OR TITLE ("overweight") OR TITLE ("obesity") OR TITLE ("BMI") OR TITLE ("body AND mass AND index")) AND (TITLE-ABS-KEY ("policy") OR TITLE-ABS-KEY ("policymakers") OR TITLE-ABS-KEY ("decision AND maker") OR TITLE-ABS-KEY ("health AND policy") OR TITLE-ABS-KEY ("HTA") OR TITLE-ABS-KEY ("Health AND technology AND assessment") OR TITLE-ABS-KEY (government) OR TITLE-ABS-KEY (authority)) AND (TITLE-ABS-KEY ("budget") OR TITLE-ABS-KEY ("medical AND expenses") OR TITLE-ABS-KEY ("healthcare AND costs") OR TITLE-ABS-KEY ("financial AND management") OR TITLE-ABS-KEY ("economics") OR TITLE-ABS-KEY ("barriers")) AND (PUBYEAR > 2013 AND PUBYEAR < 2025) AND ( LIMIT-TO ( LANGUAGE,"English" ) ) AND NOT (TITLE-ABS-KEY ( dementia ) OR TITLE-ABS-KEY ( tubercolosis ) OR TITLE-ABS-KEY ( hiv ) OR TITLE-ABS-KEY ( cancer ) ) AND PUBYEAR > 2013 AND PUBYEAR < 2025 AND ( LIMIT-TO ( LANGUAGE , "English" ) ) AND ( EXCLUDE ( EXACTKEYWORD , "Cross-sectional Study" ) AND EXCLUDE ( EXACTKEYWORD , "Randomized Controlled Trial" ) ) |
| ProQuest | 15-01-2024 | 308 | (title(Obesity) OR title(Overweight) OR title(Obese) OR title(body mass index) OR title(BMI) OR title (Adipositas)) AND (abstract(Reimbursement) OR title(Reimbursement) OR abstract(funding) OR title(funding) OR abstract(budget) OR title(budget) OR abstract(medical expenses) OR title(medical expenses) OR abstract(healthcare costs) OR title(healthcare costs) OR abstract(financial management) OR title(financial management) OR abstract(economics) OR title(economics) OR abstract(barrier) OR title(barrier)) AND (abstract(Policy) OR abstract(Policymakers) OR abstract(government) OR abstract(authority) OR abstract(Decision-maker) OR abstract(health policy) OR title(Policy) OR title(Policymakers) OR title(government) OR title(authority) OR title(Decision-maker) OR title(health policy) OR title (HTA) OR abstract (HTA) OR title (health technology assessment) OR abstract (health technology assessment)) AND NOT ((title(randomised controlled trial) NOT (title(cohort study) OR title(Randomised Control Trial) OR title(Cross-sectional study) OR title(Case-Control Study) NOT title(Cohort ))))  Limits: Publication date 2014-2024 |

Appendix 1, table 2: Search strategy in HTA databases

| **HTA database** | **URL** | **Date** | **Articles identified** | **Search terms** |
| --- | --- | --- | --- | --- |
| NICE | https://www.nice.org.uk/ | 25-1-2023 | 15 | Obesity |
| CADHT | https://www.cadth.ca/ | 29-01-2023 | 35 | Obesity |
| TLV | https://www.tlv.se/ | 29-01-2023 | 6 | Fetma |
| NT-rådet | https://janusinfo.se/ | 29-01-2023 | 0 | Semaglutid/Liraglutid  + Fetma |
| SBU | https://www.sbu.se/en/ | 29-01-2023 | 17 | Obesity |
| NIPH | https://www.fhi.no/ | 29-01-2023 | 4 | fedme |
| Nye metoder | https://www.nyemetoder.no/ | 29-01-2023 | 10 | fedme |
| Sundhedsstyrrelsen | https://www.sst.dk/da/ | 29-01-2023 | 103 | Overvægt |
| MSAC | http://msac.gov.au/ | 29-01-2023 | 15 | Obesity |
| PBS | https://www.pbs.gov.au/pbs/ | 29-01-2023 | 38 | Obesity |
| Pharma | https://pharmac.govt.nz/ | 29-01-2023 | 15 | Obesity |
